# The cardiac molecular setting of metabolic syndrome in pigs reveals disease susceptibility and suggests mechanisms that exacerbate COVID-19 outcomes in patients

**DOI:** 10.1038/s41598-021-99143-w

**Published:** 2021-10-05

**Authors:** Olivia Ziegler, Nivedita Sriram, Vladimir Gelev, Denitsa Radeva, Kostadin Todorov, Jun Feng, Frank W. Sellke, Simon C. Robson, Makoto Hiromura, Boian S. Alexandrov, Anny Usheva

**Affiliations:** 1 Division of Cardiothoracic Surgery, Department of Surgery and, The Warren Alpert Medical School, Brown University, Providence, RI 02903 USA; 2 Beth Israel Deaconess Medical Center, Harvard Medical School, Boston, MA 02115 USA; 3 Department of Chemistry, Sofia University, Sofia, Bulgaria; 4 Medical University, Sofia, Bulgaria; 5 Daiichi University of Pharmacy, Fukuoka, 815-8511 Japan; 6 Los Alamos National Laboratory, Los Alamos, NM 87545 USA

**Keywords:** Cardiovascular diseases, Transcriptomics

## Abstract

Although metabolic syndrome (MetS) is linked to an elevated risk of cardiovascular disease (CVD), the cardiac-specific risk mechanism is unknown. Obesity, hypertension, and diabetes (all MetS components) are the most common form of CVD and represent risk factors for worse COVID-19 outcomes compared to their non MetS peers. Here, we use obese Yorkshire pigs as a highly relevant animal model of human MetS, where pigs develop the hallmarks of human MetS and reproducibly mimics the myocardial pathophysiology in patients. Myocardium-specific mass spectroscopy-derived metabolomics, proteomics, and transcriptomics enabled the identity and quality of proteins and metabolites to be investigated in the myocardium to greater depth. Myocardium-specific deregulation of pro-inflammatory markers, propensity for arterial thrombosis, and platelet aggregation was revealed by computational analysis of differentially enriched pathways between MetS and control animals. While key components of the complement pathway and the immune response to viruses are under expressed, key N6-methyladenosin RNA methylation enzymes are largely overexpressed in MetS. Blood tests do not capture the entirety of metabolic changes that the myocardium undergoes, making this analysis of greater value than blood component analysis alone. Our findings create data associations to further characterize the MetS myocardium and disease vulnerability, emphasize the need for a multimodal therapeutic approach, and suggests a mechanism for observed worse outcomes in MetS patients with COVID-19 comorbidity.

## Introduction

Metabolic syndrome (MetS) and obesity are on the rise all over the world. The link between MetS and the increased risk of cardiovascular disease (CVD) is one of the most worrying consequences of this trend contributing to considerable morbidity and mortality^1^.

Numerous cases demonstrate that cardiac complications, including myocarditis, acute myocardial infarction (MI), heart failure, and arterial and venous thromboembolic events are comon in patients with MetS^2^. As part of MetS, hypertension, obesity, hyperglycemia and hyperlipidemia individually and synergistically, increase myocardial vulnerability and are associated with greater risk for death in patients with CVD; more recently components of MetS have been identified as a risk for worse COVID-19 outcomes^3^. Patients with pre-existing cardiovascular disease (CVD) and obesity have a 14% fatality rate compared to a 2.4% average COVID-19 case fatality rate^4^.

Many of the mechanisms underlying the specificity of the myocardial response to MetS, however, are still unknown. The lack of clarity results in part from the general obscurity of the molecular and metabolic processes that underlie the tissue level myocardial response to MetS. There is little data on local cross-talk between gene expression, metabolites alterations, and myocardial functionality.

Given the unique gene expression pattern and metabolic properties of the myocardium, analyses of blood or blood components are unlikely to capture the entirety of metabolic changes that the myocardium undergoes in MetS. Difficulties inherent in obtaining human myocardial tissue as well as differences in MetS treatment between humans and small animal models, further complicate the identification of the myocardium-specific risk factors in MetS.

To address these shortcomings, we intend to define myocardial variables in MetS that may contribute to an elevated risk of myocardial disease using a clinically relevant large animal model (pig) that reproducibly replicates the myocardial pathophysiology of MetS in patients^5^. Pigs have similar cardiac size, heart rate, blood pressure, and coronary anatomy to humans, and the temporal and spatial development of myocardial infarction resembles that of patients^6^. When compared to lean diet control pigs (LD), Yorkshire and Ossabaw pig fed an obesogenic high-fat-high-calory diet quickly develop the hallmark components of MetS, including: hyperglycemia, significant increases in triglycerides, plasma LDL, total cholesterol, significant weight gain, and increased systolic and diastolic blood pressure^7,8^. It has been proposed that porcine respiratory coronavirus (PRCV) infection in obese pigs be considered as highly relevant animal model for severe COVID-19^9^.

In this study, Yorkshire pigs were used as a model for high-fat-high callory diet induced MetS, along with LD control pigs^7,8^. Pig myocardium was harvested to generate gene expression profile using whole transcriptome shotgun sequencing (RNA-seq), as well as coupled proteomic and 297 polar metabolites profile using liquid chromatography–tandem mass spectrometry (LC/MS–MS). We combined proteomic, metabolomic, and physiological data with bioinformatics to distinguish genes expression profiles from MetS and LD pigs. We discovered that on a local level, the myocardium defends itself against MetS by causing severe changes in vascular integrity, platelet activation, thrombosis and fibrinolysis balance, complement activation, and the renin–angiotensin system. Our data suggest that metabolites derangements such as uric acid (UA), S-adenosyl methionine (SAM), asymmetric dimethylarginine (ADMA), ADP, hypoxanthine, and cAMP, have increased myocardial abundance in MetS. We discovered that the metabolites content of blood and cardiac tissue differed significantly, meaning that blood composition does not adequately represent cardiac response to diet. Our findings could help us better understand and anticipatee how COVID-19 affects the myocardium in patients with MetS.

Our findings add to our knowledge of the tissue-level molecular alterations that occur in the myocardium of MetS patients. Additionally, given the dysregulation of factors implicated in the viral immune response generally, as well as SARS-CoV2 specifically, these data may suggest a putative mechanism for the worse COVID-19 outcomes observed in patients with components of MetS.

## Results

### High-fat high-calory diet leads to MetS in the pig model

As we previously reported^7^, in response to twelve weeks long high-fat-high-calorie diet, five months old intact male Yorkshire pigs in our model develop key components of metabolic syndrome: increased weight gain (55 ± 2.5 kg vs 23 ± 2.7 kg), systolic (159 ± 4 mmHg vs 119 ± 3 mmHg) and diastolic (110 ± 5 mmHg vs 69 ± 12 mmHg) blood pressure, fasting plasma glucose (161 ± 15 mg/dL vs 94 ± 12 mg/dL, p < 0.02), triglycerides (1.68 ± 0.5 mmol/L vs 0.65 ± 0.24 mmol/L, p < 0.03), plasma LDL (2.69 ± 0.26 vs 0.47 ± 0.14, p < 0.01), and total cholesterol (5.7 ± 0.6 mmol/L vs 1.09 ± 0.3 mmol/L, p < 0.009I) vs regular normoglycemic diet.

Together, the phenotype observed in the pigs on high-fat, high-calorie diet meets all five metabolic syndrome diagnostic criteria in humans: obesity, elevated fasting blood sugar, elevated triglycerides and LDL, and increased blood pressure.

### MetS alters the signature of myocardial gene expression

In order to profile the myocardial gene expression in response to MetS, we performed RNA-Seq on myocardial left ventricular tissue. Tissues were isolated from four MetS and four LD (lean diet) animals within one experiment to prevent possible batch results. LC–MS/MS was performed on parallel samples from six of the pigs, three MetS and three LD, to verify RNA-seq results at the protein level. Metabolomics LC/MS–MS data derived from these same eight pigs were correlated to pathways related to the observed metabolic products.

From the RNA-Seq data, genes that show a statistically significant change in abundance between MetS and LD pigs (threshold of 1.5-fold change, p < 0.05) were selected for analysis. To perform biological processes-, pathways-related and disease interpretation of the differentially expressed MetS vs LD genes we used g:Profiler (Supplementary Figure 1). We included in the search: Gene Ontology Biological Processes (BP) database, KEGG pathways, Reactome (REAC), WikiPathway (WP), human disease phenotypes from Human Phenotype Ontology (HP). The g:Profiler revealed the following major classifications of the overrepresented biological pathways that were altered in MetS: response to stress (p_adj_ = 3.176e^−13^), hemostasis (p_adj_ = 3.371e^−11^), blood coagulation (p_adj_ = 2.768e^−11^), complement and coagulation cascades (3.108e^−14^), platelet activation (p_adj_ = 1.067e^−3^), COVID-19 disease (p_adj_ = 5.836e^−9^), venous thrombosis (p_adj_ = 1.381e^−7^). We confirmed specific transcriptomics changes for individual pigs using data from parallel LC–MS/MS proteomics and LC–MS/MS targeted metabolomic.

### MetS increases the abundance of genes with proinflammatory and oxidative stress functions

Elevated LDL, cholesterol, and hypertension are all known to induce a pro-inflammatory vascular response, findings that were recapitulated in our aforementioned metabolite and RNA-seq data.

**Figure 1:**
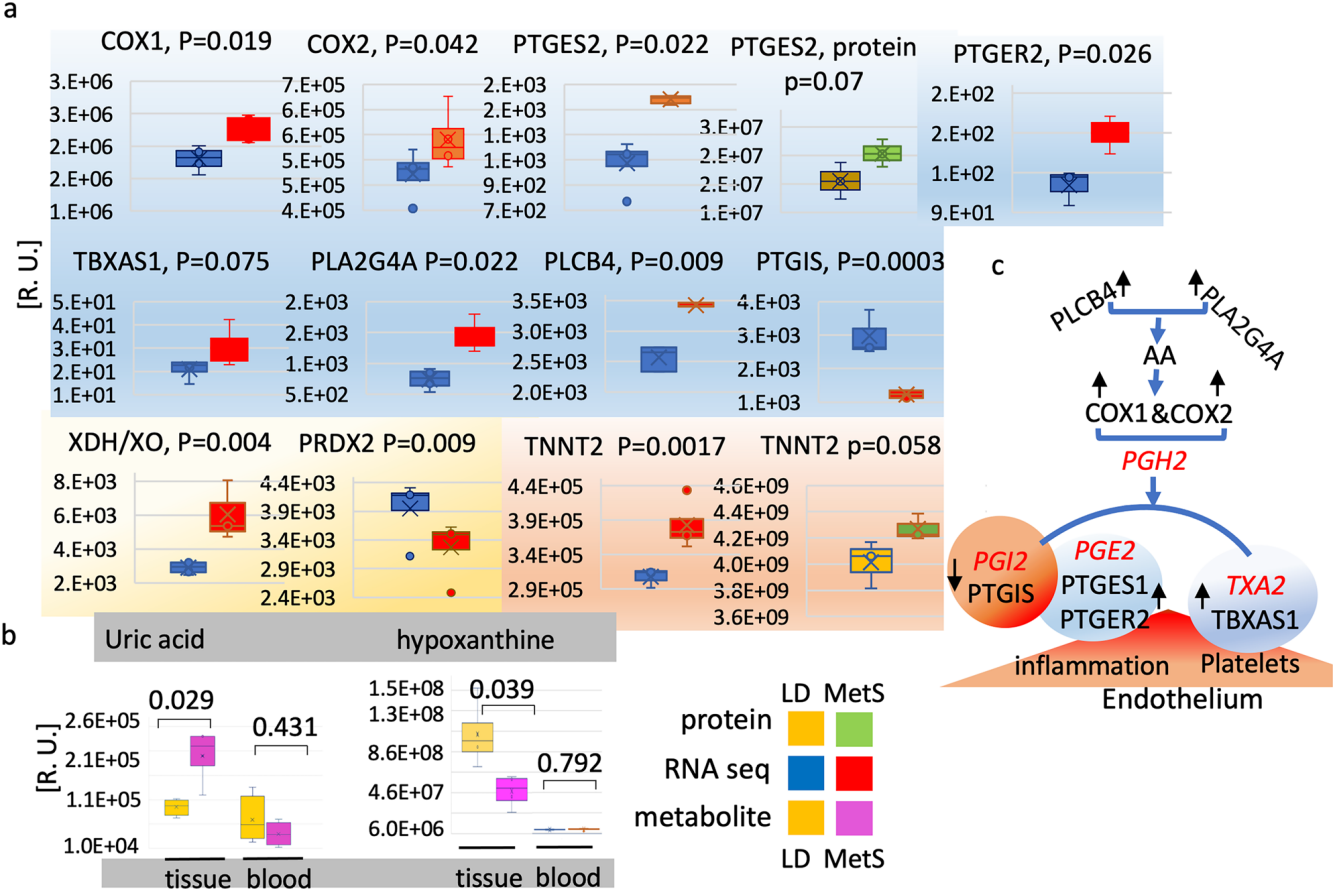
The myocardium reacts to MetS with altered expression of genes and content of metabolites associated with inflammation and oxidative stress. RNA-seq, proteomics and targeted polar metabolites LC/MS–MS were applied to identify and compare contents of gene products and metabolites that change in response to diet. Myocardial mRNA, proteomic, and targeted polar metabolites libraries from LD (n = 4) pigs and MetS (n = 4) pigs were compared for relative content of genes and metabolites that change expression in response to diet. Metabolites availability is compared in tissue and in blood (**a**) Proinflammatory factors from the arachidonic acid pathway that are significantly altered in response to diet are gated in blue: COX1, COX2, prostaglandin E synthase 2 (PTGES2), prostaglandin E2 receptor (PTGER2), thromboxane synthase 1 (TBXAS1), phospholipase A2 (PL2G4A), phospholipase C Beta 4 (PLCB4), and prostacyclin synthase (PTGIS). All factors are compared at the RNA level. PTGES2 is compared at both the protein and RNA levels. The oxidative stress related xanthine dehydrogenase, (XDH2/XO) and peroxiredoxin 2 (PRDX2) are in yellow. The RNA and protein content of the myocardial inflammation marker myocardial troponin 2 (TNNT2) is in pink. The individual factors are shown at the top of the diagrams. Values in relative units (R.U.) are mean ± SD, p ≤ 0.050, p > 0.050; LD n = 4, MetS n = 4; 2-tailed Student’s t-test. (**b**) The XDH2/XO related metabolites hypoxanthine and uric acid are gated in gray: uric acid content in the myocardial tissue (p = 0.029), in the corresponding blood sample (p = 0.431); hypoxanthine in tissue (p = 0.039) and in blood (p = 0.792) as shown below the bars. Oxaloacetate (LD, p = 0.778; MetS p = 0.914) content did not significantly alter in tissue and blood and was used as a control for the equal loading of metabolites. Values in relative units (R.U.) are mean ± SD, p ≤ 0.050, p > 0.050.; 2-tailed Student’s t-test (**c**) The Arachidonic acid pathway diagram shows the enzymes (black) that respond to diet. The single black up-arrows show upregulated enzymes and the down-arrows downregulated enzymes. The enzyme’s products are shown in red: prostaglandin H2 (PGH2), prostaglandin E2 (PGE2), prostacyclin (PGI2), and thromboxane A2 (TXA2). The effect of altered arachidonic pathway activity is shown at the bottom of the diagram: inflammation and pro-thrombotic platelet activity in the endothelium.

The RNA-seq data from four of the MetS and four of the LD pigs (Fig. 1a) demonstrate a significant increase in the arachidonic acid (AA) pathway related phospholipase A2, *PLA2G4A* (p = 0.0224) and phospholipase beta, *PLCB4* (p = 0.0092) that have the potential to supply multiple phospholipid-derived inflammatory metabolites within the cardiac endothelium; *COX-1* (p = 0.019), *COX-2* (p = 0.042), *PTGES2* (p = 0.022) together with the *PTGER2* receptor (p = 0.026), the thromboxane synthase, *TBXAS1* (p = 0.075), as well as the xanthine dehydrogenase, *XDH2/XO* (p = 0.0044). Conversely, the prostacyclin synthase, *PTGIS* (p = 0.0305) from the arachidonic pathway as well as the antioxidant defense involved glutathione peroxidase, peroxiredoxin 2, *PRDX2* (p = 0.0092) are strikingly diminished in MetS. Due to the elevated mRNA expression levels of *PTGES2,* statistical significance at the protein level was also confirmed by mass spectroscopy. Additionally, the cardiac troponin *TNNT2* is significantly elevated in MetS (RNA, p = 0.0017; protein p = 0.058) (Fig. 1a).

Actin alfa 2, *ACTA2* (RNA, p = 0.677; protein p = 0.693), desmin, (RNA, p = 0.549; protein p = 0.791), and cardiac alpha actin, ACTC1 (p = 0.592) expression levels showed no diet-related mRNA and protein content difference and acted as internal controls for the above observations and have traditionally hae been used as such in the cardiac literature (Supplementary Figue 2a). The arachidonic acid pathway related data are graphically represented in Fig. 1c.

Additionally, the metabolomic data show that the proinflammatory vascular markers and proapoptotic xanthine dehydrogenase reaction product uric acid UA (tissue, p = 0.029; blood, p = 0.431) rise significantly within the myocardial tissue while the reaction substrate hypoxanthine (tissue, p = 0.039; blood, p = 0.792) is diminished. Concomitantly, the corresponding blood samples contain significantly less UA and hypoxanthine in MetS compared to LD swine with no diet-related difference (Fig. 1b).

Hydroxyphenylpyruvate (tissue, p = 0.778) and *N*-acetyl-l-alanine (tissue, p = 0.736) content did not significantly change in tissue and were used as a control for the equal loading content of metabolites (Supplementary Figure 2b).

### Distinct expression of genes with functions in the coagulation cascade and fibrinolysis system in MetS

**Figure 2:**
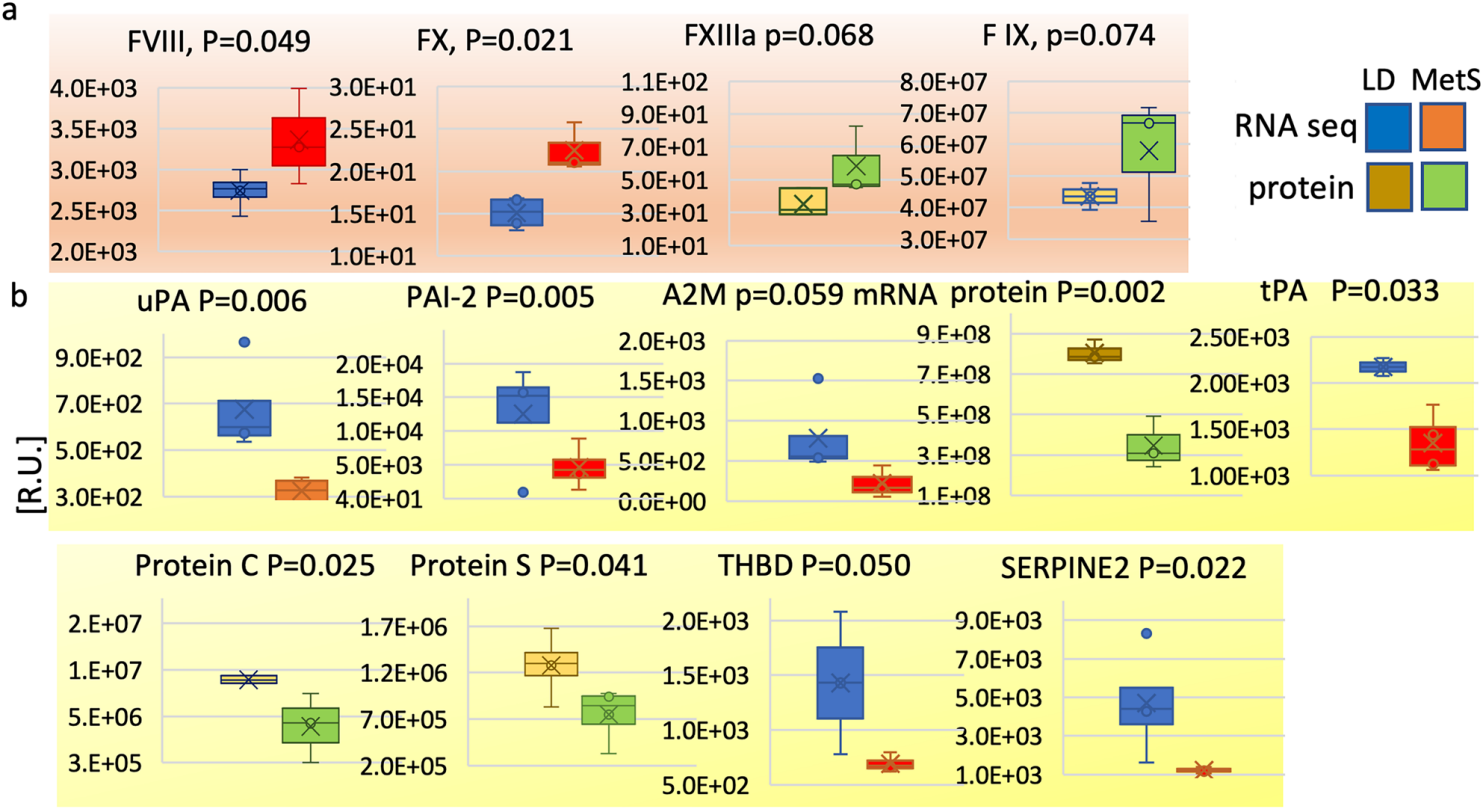
MetS related imbalance in thrombosis and fibrinolysis. Thrombosis and fibrinolysis related factors in LD and MetS myocardium are quantitatively compared by RNA-seq and protein LC/MS–MS. Data represents four LD and 4 MetS pigs. (**a**) Thrombosis related factors in MetS vs. LD are shown in pink: factor *FVIII*, factor *X*, activated factor *FXIIIa*, and factor *IX*. (**b**) Fibrinolysis related factors are shown in yellow: urokinase (*uPA)*, plasminogen activator inhibitor-2 (*PAI-2)*, alpha 2 macroglobulin (*A2M)*, tissue plasminogen activator (*tPA)*, protein *C*, protein *S*, thrombomodulin (*THBD)*, and *SERPINE2*. The identity of the genes and the p values are shown at the top of the individual diagrams. Data are presented in relative units (R.U.) as shown on the verticals; values in all bar diagrams are means ± SD; p < 0.05, p > 0.05; 2-tailed Student’s t-test; LD n = 4, MetS n = 4; RNA-seq blue (LD), red (MetS): protein bar brown (LD), green (MetS).

Given the imbalance in proinflammatory factors and the susceptibility to arterial thrombosis, it is expected that MetS myocardium will alter gene expression with roles in coagulation and fibrinolysis cascades. Therefore, the mRNA content of the genes involved in the coagulation and fibrinolysis processes in MetS and LD have been compared (Fig. 2). Some mRNA findings were recapitulated at the protein level with LC/MS–MS.

MetS myocardium shows significant increases in content of clotting effectors: factor VIII (p = 0.049), factor *X* (p = 0.021), factor IX (p = 0.062) and factor XIIIa (p = 0.059) (Fig. 2a). The RNA-seq and protein mass spec data demonstrated diminished levels of the fibrinolysis related urokinase *uPA* (p = 0.006), plasminogen activator inhibitor *PAI-2* (p = 0.005), alpha 2 macroglobulin, *A2M,* at the protein and mRNA level (protein p = 0.002, mRNA p = 0.059), protein C (p = 0.025), protein S (p = 0.041), tissue plasminogen activator *tPA* (p = 0.033), thrombomodulin *THBD* (p = 0.050) and *SERPINE2* (p = 0.022) (Fig. 2b).

Taken together, these data suggest an overall hypercoagulable state in MetS.

### MetS promotes the expression of genes with essential role in platelets activation and aggregation

Platelet involvement in myocardial complications associated with hypertension and thrombosis has been widely reported^10,11^. MetS, we believe, is linked to platelet activation and aggregation; this is most likely derived from myocardial microvasculature, which is captured in our left ventricular tissue samples.

**Figure 3:**
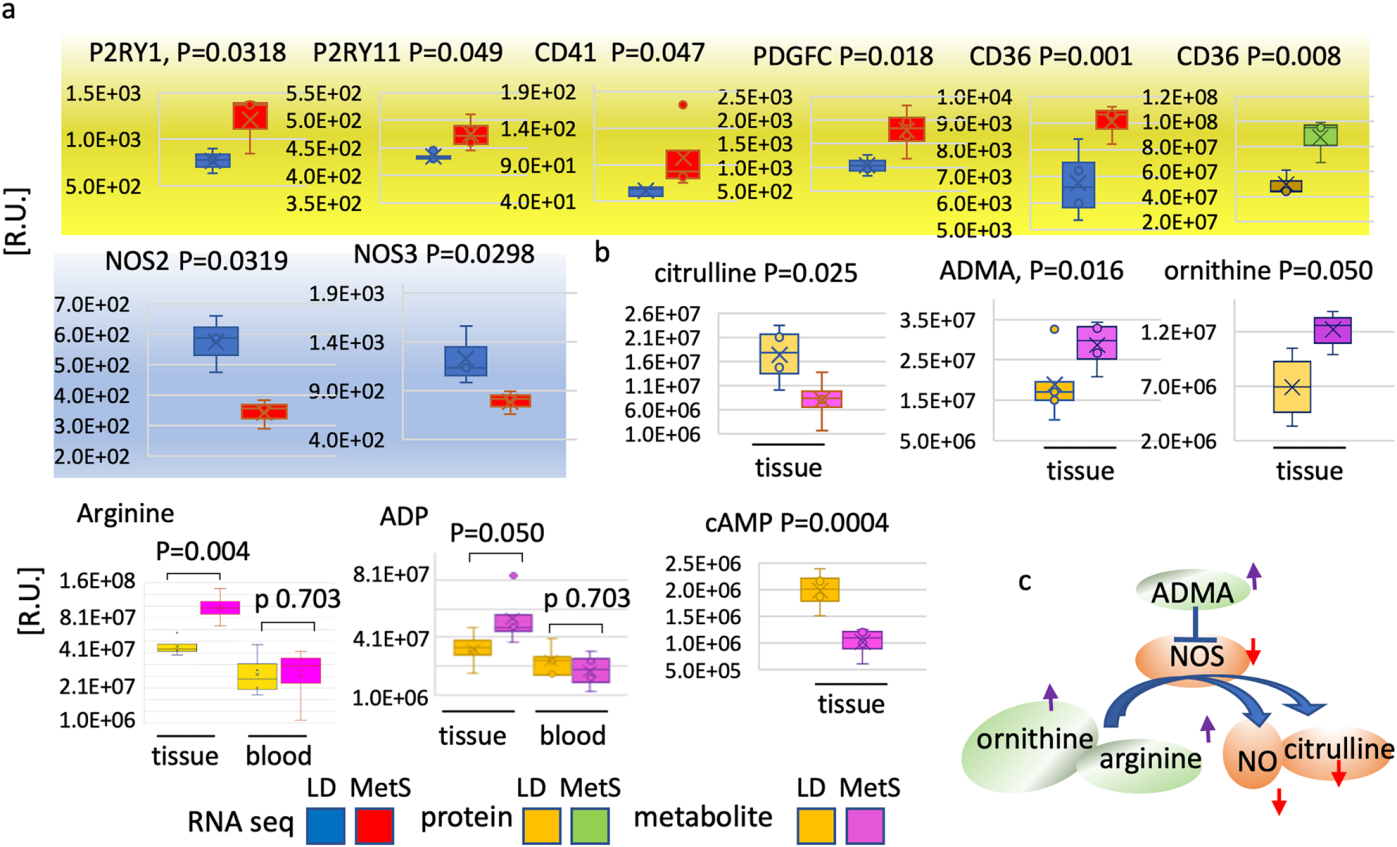
Platelet adhesion, aggregation, and activity response to LD and MetS. (**a**) RNA-seq and protein LC/MS–MS are used to quantify the diet response of genes related to platelet functions in four LD and 4 MetS pigs. Genes that change significantly in response to diet are highlighted: yellow—platelet aggregation related P2Y purinoreceptor 1 (*P2RY1)*, P2Y purinoreceptor 11 *(P2RY11)*, integrin alpha 2b (*CD41*), platelet-derived growth factor C (*PDGF-C*), platelet glycoprotein 4 (*CD 36*) at mRNA and protein level; blue- nitric oxide synthase 2 (*NOS2*) and nitric oxide synthase 3 (*NOS3*) mRNA. The gene identity and the p values are shown on top of the bar diagrams next to the individual genes’ abbreviation; mRNA bars are in blue for LD (n = 4) and in red for MetS (n = 4); data are presented in relative units [R.U] on the verticals; proteomics derived data are in yellow for LD and green for MetS; values are means ± SD; p ≤ 0.050. (**b**) NOS related metabolites are quantitatively compared by LC/MS–MS in LD and MetS heart tissue and blood, as shown below the panels. Data represents 5 LD and 5 MS pigs. The identity of the metabolites is shown at the top of the diagrams together with the p values: citrulline, arginine, ornithine, asymmetric dimethylarginine (ADMA), adenosine diphosphate (ADP), cyclic adenosine monophosphate (cAMP)—yellow bars for LD; purple bars for MetS. The individual metabolites data are presented in relative units [R.U] on the verticals; values are mean ± SD, p ≤ 0.050; data represents n = 4 LD and n = 4 MS pigs. (**c**) The NO synthesis diagram shows the metabolites that respond to diet: the upregulated in MetS vs LD metabolites are gated in green and black up-arrows; downregulated are in red and red down-arrows.

Our results show (Fig. 3a) that the MetS myocardium displays a substantial rise in the platelet activation related receptors *P2RY1* (p = 0.0318) and *P2RY11* (p = 0.049), platelet-derived growth factor *PDGFC* (p = 0.018), which is associated with the ADP-promoted platelet aggregation αIIbβ3 integrin, *CD41* (p = 0.050) and the platelet glycoprotein 4, *CD36* (mRNA p = 0.001, protein p = 0.008).The mRNA expression level of v*VWF*, and the platelet activation related receptors *P2RY12* and *P2RY6,* were not significantly altered between MetS and control animals and served as an internal control for the observed differences (Supplementary Figure 2a). The level of CD36 was shown by Western blot as well (Supplementary Figure 2e).

Importantly, the endothelial specific thrombo-regulators and inhibitors of platelets reactivity endothelial nitric oxide synthase *eNOS* or *NOS3* (p = 0.0249) and the inducible *iNOS or NOS2* (p = 0.0319) are noticeably decreased in MetS as well. The metabolomic results also show altered content of metabolites that are involved within the nitric oxide (NO) synthesis by NOSs (Fig. 3b). Although the availability of NO precursors, l-arginine (p = 0.0039) and ornithine (p = 0.050), in MetS is substantially higher than in LD, the ultimate NOS reaction product l-citrulline (p = 0.0247) is significantly reduced, implying that NO availability is also reduced. Asymmetric dimethylarginine, another l-arginine derivative and NOS inhibitor is significantly increased in MetS (Fig. 3b). ADP, a significant promoter of platelet aggregation, is also higher in MetS vs LD (p = 0.050), while cAMP is greatly reduced (p = 0.004). The metabolomics data related to the NO synthesis are graphically represented in Fig. 3c.

Again, as ADP is considerably more abundant in tissue than in blood, this suggests a myocardial-specific pro-inflammatory environment. Taken together, these data support the hypothesis that MetS promotes the expression of genes with significant roles in the activation and aggregation of platelets. Importantly, these results suggest vascular accumulation and aggregation of platelets, which are the central mediators of thrombosis.

### The innate immune system responds to MetS

**Figure 4:**
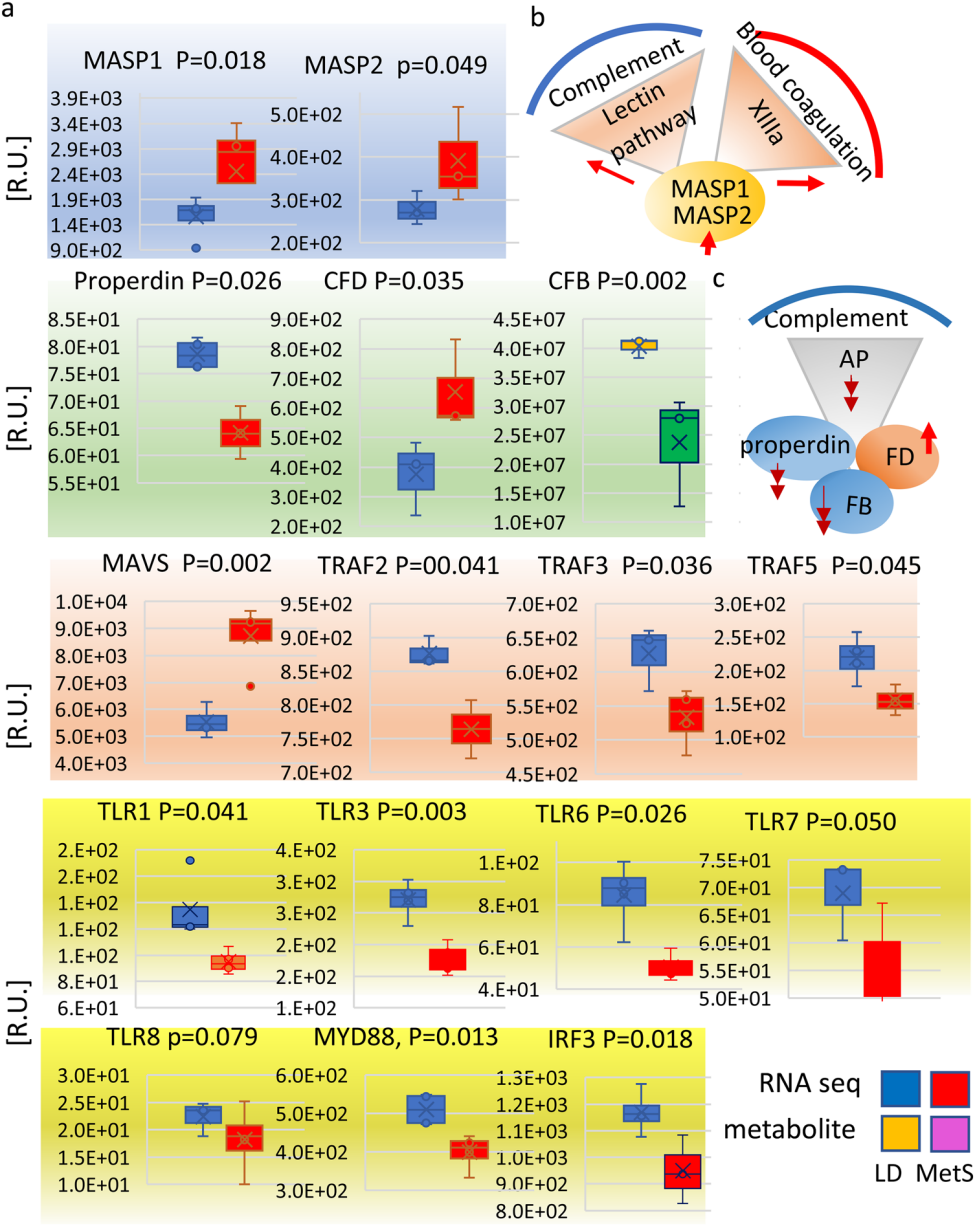
Innate immune system response to MetS. (**a**) The lectin pathway, the alternative pathway, and the coordinators of the antiviral innate immunity in the MetS and LD myocardium are identified and quantitatively compared by RNA-seq and proteomic LC/MS–MS as indicated. Data represents 4 LD and 4 MetS pig. The lectin pathway related factors are gated in blue: mannan-binding lectin serine protease 1 (*MASP-1* and mannan-binding lectin serine protease 2 (*MASP-2*). The alternative pathway (AP) complement factors are gated in green: properdin, complement factor D (*CFD*), complement factor B (*CFB*). The coordinators of the antiviral innate immunity are gated in pink: the mitochondrial anti-RNA virus infection signaling protein (*MAVS*), the TNF receptor-associated factor 2 (*TRAF2*), TNF receptor-associated factor 3 (*TRAF3*), TNF receptor-associated factor 5 (TRAF5); the Toll-like receptors TLR1, TLR3 TLR6, TLR7, TLR8, together with the interferon regulatory factor 3 (*IRF3*) are gated in yellow. The gene identity and the P values are shown on top of the bar diagrams next to the individual genes’ abbreviation. The RNA-seq bar diagrams are labeled in blue for LD and red for MetS, while the proteomics related bar diagrams are in brown for LD and green for MetS. Data are presented in relative units (R.U.) as shown on the verticals. The values in all bar diagrams are means ± SD; p = 0.050. (**b**) The diagram represents the simultaneous involvement of MASP1 and MASP2 in the lectin pathway and in the blood coagulation by activating factor XIIIa; red arrows—significant activation in MetS. (**c**) The alternative pathway (AP) diagram shows the gene products that respond to diet; red double arrows-significant decrease; red single up-ward arrow—increased expression in MetS vs. LD.

As part of the innate immune system the complement system is known to cooperate with toll like receptors (TLRs) as a defense against inflammation and in response to obesity, hyperglycemia, and insulin resistance^12^. In order to find a possible response mechanism of the myocardial innate immune system in MetS, we compared the mRNA content of genes involved in both, the complement system and in the innate immune system (Fig. 4).

Highly expressed in MetS are mRNAs for the lectin complement pathway related serine proteases *MASP-1* (p = 0.018) and *MASP-2* (p = 0.049) while the expression of properdin (p = 0.026) and factor B, *CFB* (p = 0.002) from the alternative complement pathway (AP) are significantly diminished and factor D, *CFD* (n = 4, p = 0.035) is highly expressed (Fig. 4a). Furthermore, the protein content of complement *C1qBP* and *C1QA* did not differ between MetS and control animals, serving as an internal control for the observations made above (Supplementary Figure 1a). The antiviral innate immune system related mitochondrial common adaptor protein *MAVS* (p = 0.002) is highly expressed in MetS, whereas tumor necrosis factor (TNF) receptor-associated factors *TRAF2* (p = 0.041), *TRAF3* (p = 0.036), and *TRAF5* (p = 0.041) are significantly reduced. Toll like receptors *TLR1* (p = 0.0431), *TLR3* (p = 0.00356), the myeloid *TLR6* (p = 0.026), *TLR7* (p = 0.050), *TLR8* (p = 0.079), and the TLR downstream and anti-viral regulatory factor 3 *IRF3* (p = 0.018) are significantly reduced in MetS. Figure 4b,c show simplified representations of the lectin pathway coagulation and the alternative pathway (AP) factor involvement in MetS.

These data support the notion that in MetS, the myocardial anti-inflammation and anti-viral protection are severely diminished.

### The RNA methylation potential in MetS is elevated

**Figure 5:**
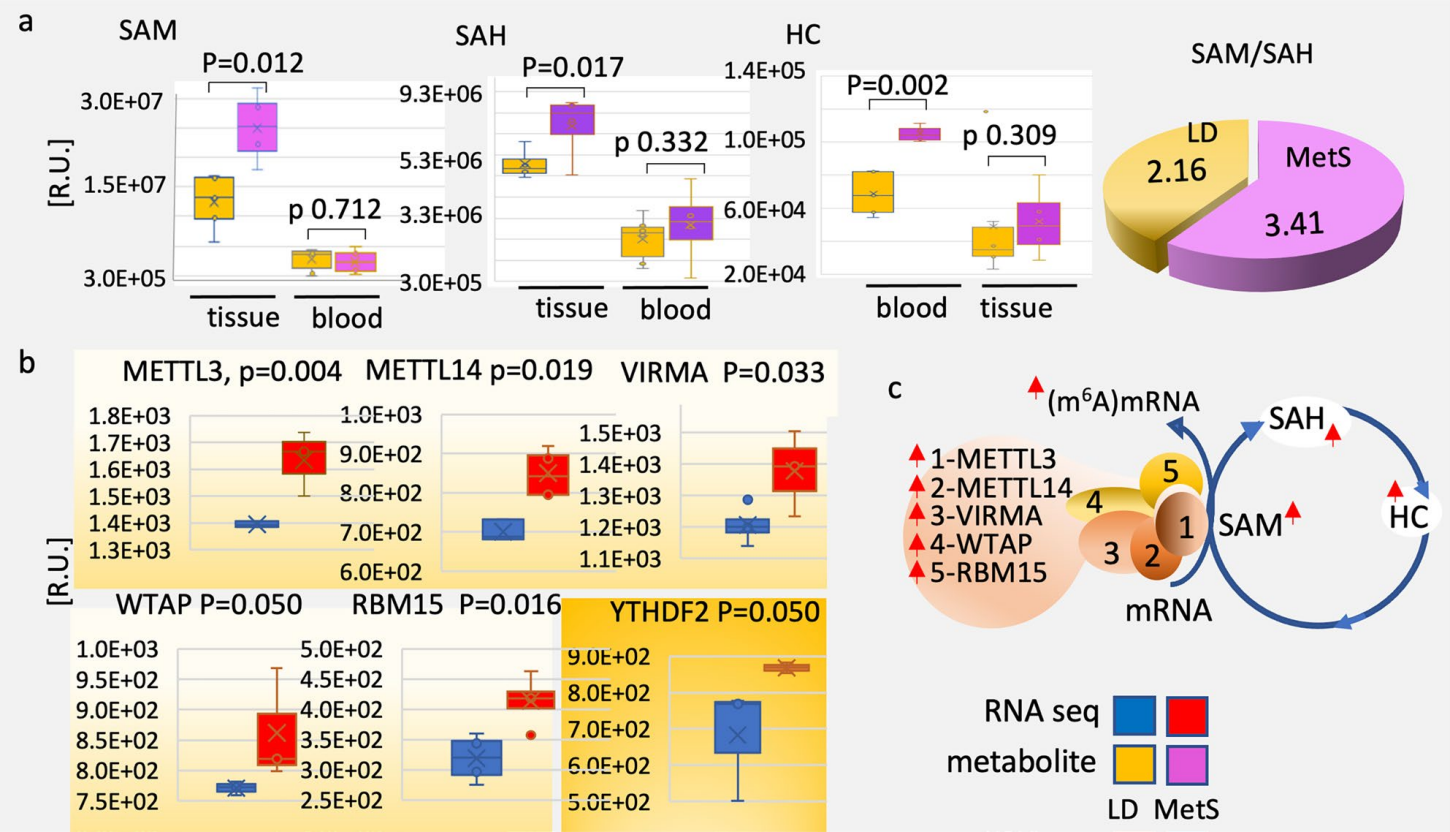
The (m6A) RNA modification complex’s responds to MetS. (**a**) Metabolites that are essential for N6-(m6A) mRNA methylation are quantitatively compared in MetS vs LD by LC/MS–MS: S-adenosyl methionine (SAM), S-adenosyl homocysteine (SAH), homocysteine (HC). The metabolite identity and the p values are shown on top of the bar diagram-yellow bars for LD and purple bars for MetS. Metabolites are extracted and analyzed from the myocardial tissue and blood of the corresponding pigs as shown below the bars for LD (n = 5) and MetS (n = 5) pigs. The values in all bar diagrams are means ± SD; p = 0.050. The 3-D Pie chart presents the SAM/SAH ratio of content: LD-yellow (2.16 ± 0.35), MetS-purple (3.41 ± 0.21). (**b**) In yellow are gated overrepresented in MetS vs LD myocardial genes that achieve the (m6A) methylation of mRNA: methyltransferase-like 3 (*METTL3*), methyltransferase-like 14 (*METTL14*), the regulatory Wilms tumor suppressor-1 (*WTAP*), the N6-methyladenosine (m6A) methylation mediator of RNAs (*VIRMA*), the RNA-binding protein 15 (*RBM15*), and the RNA stability regulator YTH domain-containing family protein 2 (*YTHDF2*). The color identity of the LD and MetS bars is shown on right. The gene identity and the P values are shown on top of the bar diagrams next to the individual genes’ abbreviation. Data are presented in relative units (R.U.) as shown on the verticals. The values in all bar diagrams are means ± SD; p ≤ 0.050. (**c**) The N6-(m6A) mRNA methylation diagram shows the metabolites and the gene products that respond to diet; red arrows—significant increase in MetS.

The N6-methyladenosine (m6A) modification of mRNA, which regulates mRNA stability, localization, and translation has been linked to cardiac pathologies and viral infections^13,14^. The response of the (m6A) mRNA modification complex’s to MetS is unknown. Our targeted metabolomics data show that the main methyl donor for (m6A)RNA, S-adenosyl methionine, SAM (n = 5, p = 0.012), its conversion products after methyl transfer, S-adenosyl homocysteine SAH (n = 5, p = 0.017), and homocysteine HC (p = 0.002) as well as the SAM/SAH ratio (LD 2.16 ± 0.12; MetS 3.41 ± 0.23) have increased significantly (Fig. 5a). Concurrently, MetS blood samples contain significantly less SAM, SAH, and HC in MetS than LD swine, with no diet-related difference (Fig. 5a).

**Figure 6:**
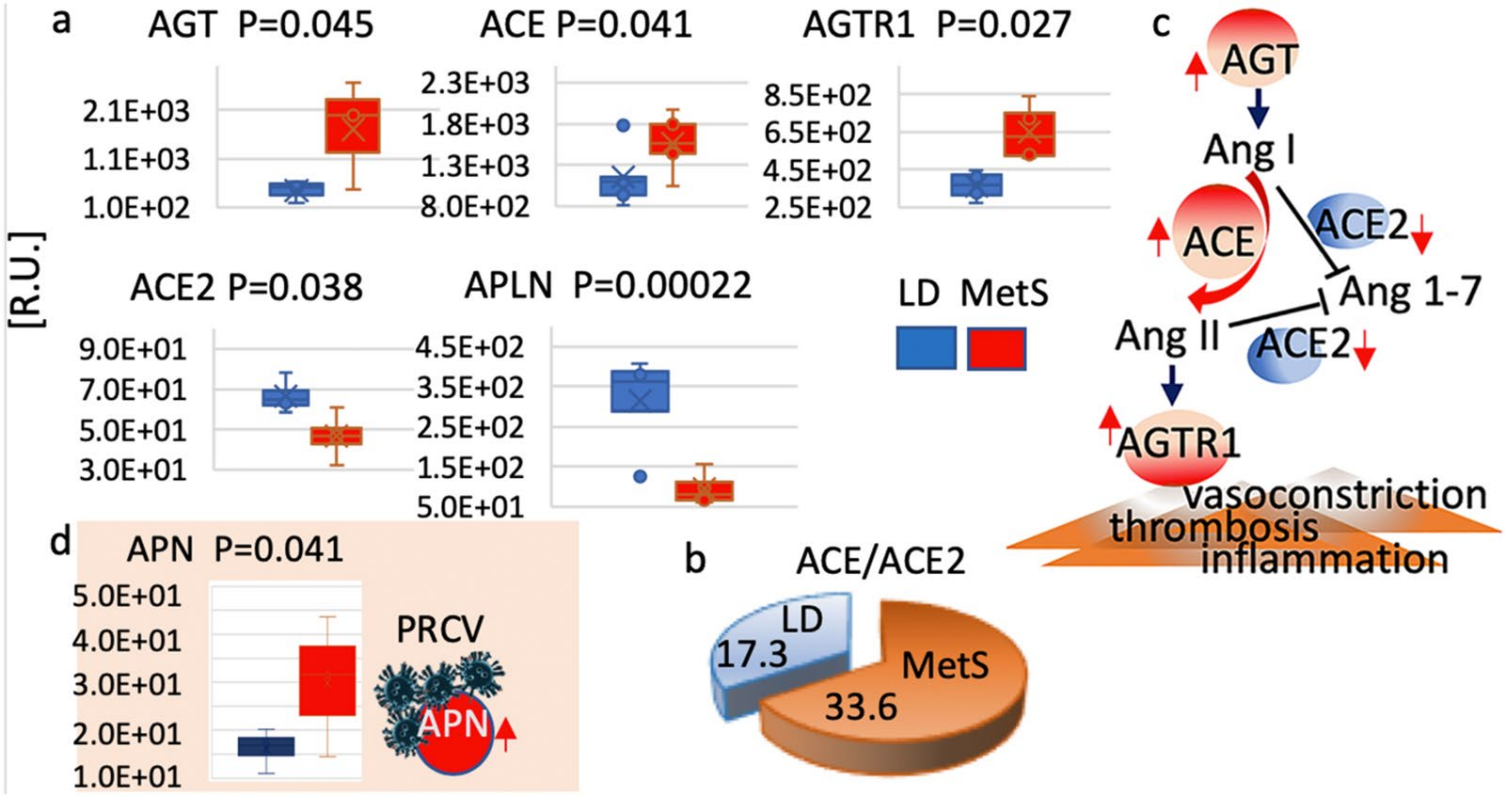
Changes in expression of genes with functions in the RAAS system. (**a**) Genes with essential functions in RAAS and RNA-seq identified MetS vs LD changes in expression: angiotensin receptor (*AGTR1*), angiotensin (*AGT*), angiotensin converting enzyme (*ACE*) and (*ACE2*), apelin (*APLN*). The RNA-seq bar diagrams are shown in blue for LD (n = 4 pigs) and red for MetS (n = 4 pigs); the gene identity and the P values are shown on top of the bar diagrams next to the individual genes’ abbreviation; data are presented in relative units (R.U.) as shown on the verticals; values in all bar diagrams are means ± SD; p = 0.050. (**b**) The pie chart presents the ACE/ACE2 ratio of mRNA content: blue for LD (17.3 ± 3.1) and brown for MetS (33.6 ± 2.5). (**c**) The RAAS pathway diagram shows in pink the genes that are significantly upregulated in MetS; downregulated in MetS are in blue. The single red up-arrows show upregulated enzymes; double red down-arrow show downregulated enzymes. (**d**) The porcine respiratory coronavirus (PRCV) receptor aminopeptidase N (*APN*) expression in LD and MetS (p = 0.041); the diagram on right represent the PRCV binding to ANP.

Furthermore, in MetS, the catalytic subunits of the highly conserved methyltransferase complex, methyltransferase-like 3, *METTL3* (p = 0.004) and methyltransferase-like 14, *METTL14* (p = 0.019) as well as the regulatory subunits Wilms tumor suppressor-1, *WTAP* (p = 0.050), *VIRMA* (p = 0.033), and RNA-binding protein 15, *RBM15* (p ≦ 0.016) are all noticeably increased in MetS (Fig. 6b). The YTH domain-containing family protein 2 ‘reader’, *YTHDF2* (p = 0.050) is also significantly elevated in MetS. Figure 5c depicts a simplified version of the m6A mRNA methylation and the factors that are altered in MetS.

According to the findings, activating key metabolic and protein regulators of myocardial and viral (m6A) RNA methylation promotes mRNA translation and stability in MetS.

### The renin–angiotensin–aldosterone cascade response to MetS

The renin–angiotensin–aldosterone system (RAAS) regulates blood pressure and systemic vascular resistance, and it has recently been shown to mediate myocardial SARS-CoV-2 infection through the angiotensin-converting enzyme 2 (ACE2) receptor^15^. Although RAAS serves an important function, in MetS it can be activated inappropriately, contributing to the hypertension seen as part of the disease. Using RNA-seq data sets, we compared the abundance of key RAAS system components in LD and MetS (Fig. 6a). MetS data show a statistically significant increase in angiotensinogen *AGT* (p = 0.045), angiotensin converting enzyme *ACE* (p = 0.041), and angiotensin type 1 receptor A*GTR1* (p = 0.027). *ACE2* (p = 0.038) and the relatively abundant apelin, *APLN* (p = 0.0002) showed significant decrease in MetS. Furthermore, the ACE/ACE2 ratio is significantly higher in MetS than in LD (Fig. 6b). Figure 6c depicts simplified graphical version of the RAAS data. While the human coronavirus SARS-CoV-2 receptor ACE2 is low, the RNA-seq results show that the receptor for the pig respiratory coronavirus (PRCV) aminopeptidase N (APN) (p = 0.042) was substantially increased in MetS (Fig. 6d)^16^.

The RAAS signaling abnormalities point to a mechanism for an increase in hypertension and subsequent hypertrophy. The high aminopeptidase N suggests hight propensity for PRCV entry.

**Figure 7:**
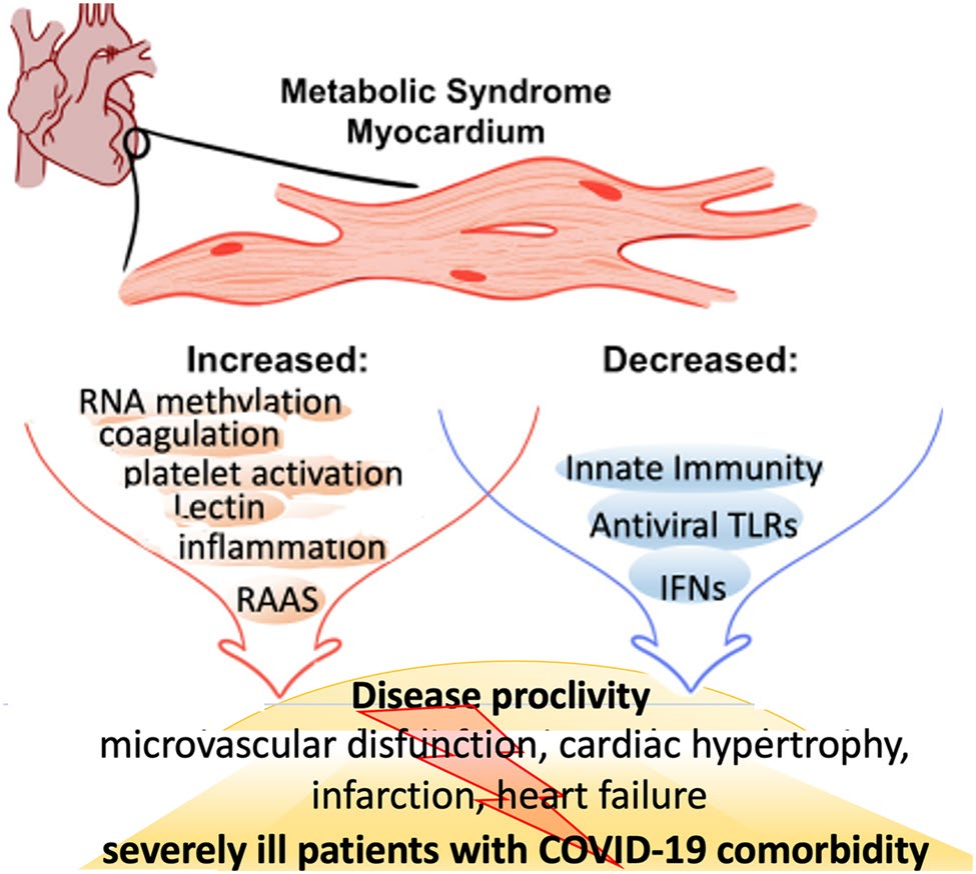
Graphical abstract of the myocardial pathologic features in MetS. The pathways that are significantly elevated in MetS are gated in red; the pathways and metabolites that are significantly diminished in MetS are gated in blue. The phenotypic changes contribute to microvascular disfunction, atherosclerosis of the coronary artery increased cardiac hypertrophy, myocardial infarction, heart failure, worse COVID-19 outcome (gated in yellow).

The myocardial MetS environment is graphically summarized in Fig. 7.

## Discussion

Our novel experimental and computational strategy for simultaneously profiling myocardial genomics, proteomics and metabolomics in the pig MetS model is a significant step toward better understanding the specific derangements that occur in the MetS myocardium and may underpin the link between MetS and increased risk for cardiovascular events. Notably, the metabolic and genomic derangements found in the MetS myocardium are associated with a pro-inflammatory, pro-thrombotic state, as well as modifications in immune response components that could imply higher viral susceptibility. Of note, a putative mechanism by which MetS changes the translation of viral and endogenous genes in the myocardium is its predicposition for (m6A)RNA methylation. Other alterations in the AA and RAAS systems, specifically COX2, prostaglandin synthase-2, and the angiotensin receptor in MetS, have also been linked to CVD. Furthermore, our findings shed light on processes that contribute to the recently observed increased vulnerability to COVID-19 in patients with MetS components.

### MetS is associated with increased inflammatory mediators and oxidative stress

The arachidonic acid release catalysts phospholipase beta and phospholipase A2 have increased significantly in MetS. Many proinflammatory factors and platelet activators (broadly classified as cyclic endoperoxides) require arachidonic acid. COX2, which converts arachidonic acid into these cyclic endoperoxides, is likewise increased in the MetS myocardium. MetS increases the activity of enzymes downstream in the arachidonic acid metabolism cascade that produce PGE2 and TXA2. Overall, this indicates that in MetS, the arachidonic acid cascade is highly active, resulting in a considerable elevation of pro-inflammatory factors, platelet aggregation, and atherosclerosis^17,18^. The activation of COX2 and prostaglandin synthase type-2 in the arachidonic acid cascade has recently been discovered as viral infection target. Indomethacin therapy, which inhibits prostaglandin synthesis, is currently being tested to combat COVID-19 inflamation^19,20^.

MetS also lacks peroxiredoxin 2, an antioxidant enzyme that has been linked to the development of atherosclerotic plaques^21^. Xanthin oxidase is also up significantly in MetS. Purine metabolism necessitates xanthin oxidase for the oxidation of hypoxanthine and xanthine and the generation of uric acid. These products are produced in tandem with reactive oxygen species resulting in decreased endothelial vasodilation, which has been linked to hypertension and heart failure development^22^. The most potent of these metabolites, uric acid, has been related to acute and chronic inflammation, smooth muscle proliferation, and metabolic syndrome development^23^.

### MetS is associated with a hypercoagulable state

In addition to the direct negative effects of the proinflammatory condition in MetS, this inflammation changes the coagulation cascade. Increases in factors VIII, IX, X, and XIIIa are seen in our MtS animals, indicating clotting disruption. The coagulation cascade has been extensively researched, and elevation of certain factors has been linked to an increased risk of clotting, even when studied in isolation^24^. MetS increases clotting factors but decreases fibrinolytic proteins C and S, which work directly on factor Va and X^25,26^. Hypercoagulable disorders, arterial thromboembolism, and myocardial infarctions have all been associated to a lack of these factors^25^. Furthermore, tPA, a fibrinolytic, as well as factors that block fibrinolysis, such as PAI-2, SERPINE2, and uPA have all decreased significantly.

Given the imbalance between clotting and fibrinolysis, these findings suggest that MetS patients are predisposed to coronary thrombosis. This, combined with an already faulty MetS-related clotting mechanism, could explain why patients with MetS components have worse COVID-19 comorbidity outcomes.

### MetS is associated with platelet activation

Platelet activation is promoted by MetS hypertension, hypercholesterolemia, thrombotic conditions, arachidonic acid metabolism, COX, and xanthine oxidase activation. Platelet aggregation in MetS may be aided by elevated ADP levels and the receptor P2Y1. In MetS, PDGFC, CD36, and CD41 expression all increase, indicating a proclivity for platelet activation^27^. MetS also reduces the epression of prostacyclin synthase, which inhibits the production of prostacyclin, a potent vasodilator and platelet aggregation inhibitor.

In the MetS myocardium, the cell wall associated eNOS and the mainly cytosolic and cytokine-inducible iNOS are dramatically reduced. Impaired NOSs pathways negatively affect collateral circulation in the left ventricle. NO, the most powerful endogenous vasodilator and inhibitor of platelet adhesion to the vascular wall, is produced by these enzymes by consuming arginine. One possible reason for this suppression is asymmetric dimethyl arginine, which is elevated in MetS and has been identified as a mediator of endothelial dysfunction and reduced NO production^28^. Reduced NO bioavailability in MetS could possibly be a response to endothelial damage, resulting in the increased vascular resistance that we observed in our pig model. Furthermore, the decreased availability of iNOS, reduces NO availability and protective functions within the immune system, including the antimicrobial and antiviral defense^29^.

### Toll-like receptors and other viral immunity components are deranged in MetS

The toll-like pathogen-associated immune receptors constitute the first line of myocardial defense against invading pathogens and endogenous danger molecules produced in ischemic myocardial injury and dying cells^30,31^. In MetS, TLRs are down. MetS leads to a significant reduction in the intracellular TLR1, 3, 6, 7 and 8. TLR3 recognizes double-stranded RNA (dsRNA), whereas the two functionally overlapping TLR7 and TLR8 recognize single-stranded RNA (ssRNA) and have emerged as important therapeutic targets for retroviral infections^32^. Low TLR7 and TLR8 levels in MetS and COVID-19 comorbidity may reduce myocardial capacity to generate an antiviral response and thus blunt the immune response. TLR1 and TLR6, which have been linked to viral and bacterial infection, are also reduced^33^. The cell surface located TLR2, TLR4, and TLR10, on the other hand, show no change.

Downstream of TLRs acts IRF3, the transcriptional activator that promotes the expression of interferons alpha and beta. In MetS and critically ill patients with COVID-19 comorbidity, reduced IRF3 availability, combined with transcription factor YY1 O-glycosylation could partly, explain the impaired expression of interferons beta, and gamma^7,34–36^. The myocardium’s first line of defense against viral infection-related myocarditis appears to be dysregulated in MetS^37^.

Furthermore, because MAVS, an anti-viral signaling protein and central adaptor in the innate immune response, is significantly elevated, the MetS myocardium is especially vulnerable to viral infections and may be associated with elevated viral load capacity^37^. Additionally, we see a decrease in the availability of the MAVS downstream mediators in MetS, specifically the E3 ligases TRAF3, TRAF 2, and TRAF 5. These ligases play an essential role in the the antiviral immune response by activating IRF3 and inducing interferons. TRAF3 and TRAF 2 have recently gained attention as factors in a variety of pathological events, including inflammation and apoptosis in ischemic injury, as well as NF-kB pathway regulators^38^. Low TRAF3/IRF3 levels are likely to play a role in the poorer outcomes seen in COVID-19 patients with MetS components^39^.

### MetS: a potential link between complement and clotting factors

The complement system is an important component of the humoral immune system that functions in tandem with toll-like receptors to protect against bacterial and viral invaders, myocardial infarction, and myocardial inflammatory damage. A growing body of evidence suggests that abnormal complement activation contributes to the development of vascular complications in hyperglycemic patients. Triggered by the condition in MetS, the two known complement proteases MASP-1 and MASP-2 that function to convert complement system components to active mediators, are both significantly elevated. Both proteases act as a link between the complement and the coagulation cascades. Their rise is directly related to the rise of factor XIIIa seen in MetS. MASP-2 may also be able to functionally avoid factor Xa by directly forming fibrin, which is required for cloth formation.

MetS components have the potential to activate multiple cloth formation pathways. Properdin and factor B, both of which act as stabilizers and promoters of the alternative complement pathway, are decreased in MetS, while factor D is increased. Properdin deficiency has been linked to increased morbidity and mortality in microbial infections and factor D inhibitors may aid in the prevention of complement-mediated damage in MetS patients with COVID-19 comorbidity^40,41^.

### Features of MetS RNA methylation may enhance viral infectivity

According to our findings, MetS can activate m6A RNA methylation. MetS-associated increase in (m6A) NA methylation is suggested by the significantly increased content of the m6A methyltransferase complex partner proteins METTL3, METTL14, WTAP, VIRMA, and RBM15^42^. This increased methylation is accompanied by significantly higher levels of the methyltransferase substrate SAM, whereas the demethylases remain unchanged. SAM was recently identified as an O-methyltransferase (NSP10-NSP16 complex) methyl donor linked to COVID-19, in which m6A methylates the viral mRNA to increase translation and render it invisible to the host immune system^43^.

The N6-methyladenosine (m6A) modification, most common and complex modification of eukaryotic RNA, is a key player in host–pathogen interactions^44^. The degree and pattern of RNA m6A methylation can influence the fate of target transcripts, and cardiac hypertrophy and failure have been linked to significant changes in (m6A) mRNA^45^. While specific methylation patterns must be investigated further, this overall methylation proclivity may point to a possible mechanism by which MetS alters gene translation in the myocardium.

Infection with the highly pathogenic HIV-1 ssRNA virus and the highly pathogenic Zika, West Nile, and hepatitis C positive-sense RNA viruses has been shown to result a massive increase in host and viral m6A RNA^46^. Similar to SARS-Cov-2, our findings highlight the context of MetS as a potential advantage for viral metabolism.

### MetS is associated with derangements in the RAAS system

In MetS patients, the renin–angiotensin system (RAAS) is thought to play a local role as a homeostatic regulator of vascular function. MetS is distinguished by abnormal RAAS activation as well as elevated oxidative, inflammatory, and prothrombotic status. The activity of ACE2, which protects against the deleterious effects of the significantly elevated AGT, ACE, and AGTR1, is substantially reduced. The significantly higher ACE/ACE2 ratio observed in MetS correlates well with the increased systolic blood pressure and hyperglycemia in our pig model. Reduced ACE2 impairs the ability to balance and offset ACE, resulting in left ventricular remodeling and abnormal myocardial function. The observed apelin deficiency, which acts as a transcriptional activator of ACE2 expression, could explain the decline in ACE2 in MetS^47^. In the context of MetS, ACE2 deficiency, combined with a significant increase in ACE and AGTR1, would impair the imbalance between the ACE-angiotensin II-AGTR1 (AT1) receptor axis and the ACE2-angiotensin1-7 axis. This type of dysregulation would greatly facilitate the progression of vasoconstriction, inflammation and thrombosis, all of wich contribute to cardiac weakness and poor outcome seen in COVID-19 patients.

The human SARS-CoV-2 virus enters cells mainly via ACE2. Despite the fact that the viral spike protein and pig ACE2 bind with high affinity, the virus does not replicate or cause disease in pig. It appears likely that low ACE2 content, combined with high ACE and altered ACE/ACE2 ratio, could partly protect the pig myocardium from SARS-CoV-2 invasion^48^. In the pig myocardium, the PRCV receptor aminopeptidase N is expressed, and its expression is considerably higher in MetS, indicating a higher risk of cardiac PRCV infection. PRCV infection in pigs has been recommended as a model to investigate SARS since the pathogenesis of PRCV infection in pigs is comparable to that of SARS in people^9,49^.

Overall, our strategy for profiling myocardial genomics, proteomics, and metabolomics in a MetS pig model is an important step toward better understanding the specific molecular derangements associated with the MetS myocardium and the increased risk of cardiovascular events. The myocardial left ventricle is the environment for collateral development. Profiling the molecular and signaling changes will have a significant impact on developing new and successful strategies for vessels grow and development. Blood may not have a substantial direct impact on the molecular profile although diabetes and hyperglycemia can affect angiogenesis. One limitation of this original study is the relatively short time frame of the obesogenic diet, which may limit the long-term effects of MetS in this model. Furthermore, the response to diet and MetS could be a gender and age specific and we investigated only young, intact male animals. This model differs from that observed in human insofar as MetS may take years to develop in many individuals. However, given increasing the propensity for MetS to develop in younger patients, as well as the practical limitations on housing animals for years, our model represents a strong facsimile of MetS. This is especially true given the replication of all the components (hypertension, hyperlipidemia, hyperglycemia) of MetS with our model. While not perfect, the pig provides a versatile model that can be adjusted to mimic a variety of diets and disease scenarios^8^. Additionally, although the applied targeted metabolomics here accurately monitor only a limited number of selected polar metabolites, a protocol has been established to extract, simultaneously, detect, and compare > 900 polar and nonpolar metabolites in myocardial tissues that may give more information in the future^7^.

The current findings suggest a possible mechanism for the poor outcomes experienced by patients with MetS and COVID-19 comorbidity. Our findings could provide a molecular foundation for the development of new therapeutic targets and early interventions for cardiovascular complications in the increasingly common MetS and COVID-19 comorbidity.

## Materials and methods

### Animal model

Four to six weeks old male intact Yorkshire swine (n = 4) (Parsons Research, Amherst, MA) were fed an obesogenic 2248 kcal/day (hypercholesterolemic) diet daily: 500 g of feed composed of 4% cholesterol, 17.2% coconut oil, 2.3% corn oil, 1.5% sodium cholate and 75% regular chow (Sinclair Research, Columbia, MO) for 12 weeks and was used to model MetS. Control LD animals (n = 4) were given regular chow (1824 kcal/day) as previously reported^9^. After 12 weeks, swine were anesthetized and physiologic measurements were taken, followed by euthanasia via exsanguination. Tissue samples from all animals were taken from identical myocardial left ventricular territories (LV) and were rapidly frozen in liquid nitrogen. Blood samples were drawn (for serum isolation in red topped tubes, Becton Dickinson (BD); for plasma isolation in EDTA lavender top tubes) from the jugular vein prior to euthanasia and tissue harvest. The blood samples (red topped tubes) were incubated at ambient temperature for 30 min and centrifuged at 2000×*g* for 10 min to separate the serum from the blood clot. The serum samples for polar metabolites isolation were rapidly frozen (0.5 mL aliquots) in liquid nitrogen. The chemistry laboratory at the Rhode Island Hospital, Providence, RI, analyzed the plasma samples (lavender top tubes) for content of glucose, triglycerides, plasma LDL, total cholesterol. All analyses are conducted in agreement with the biosafety regulations at the Rhode Island Hospital, Providence, RI. All methods were carried out in accordance with relevant guidelines and regulations.

The study was carried out in compliance with the ARRIVE guidelines.

All experiments were approved by the Institutional Animal Care and Use Committee of the Rhode Island Hospital. Animals were cared for in compliance with the “Principles of Laboratory Animal Care” formulated by the National Society for Medical Research and the “Guide for the Care and Use of Laboratory Animals” (NIH publication number 5377-3, 1996).

### RNA-seq

Total RNA was extracted from fresh frozen left ventricular myocardial tissue and RNA isolated as previously reported^9^. All eight samples (4 MetS and 4 LD) submitted for sequencing (eight independent RNA isolations and samples) had RNA integrity ≥ 9.8. Libraries (50 mg/sample) were sequenced with 2 × 50 bp paired-end reads on an Illumina HiSeq 2500 in high-output mode to an average range dept of 86 × 10^6^ paired-end reads/sample, with a range of 43 × 10^6^–139 × 10^6^ at GENEWIZ (South Plainfield, NJ); reads were mapped to the porcine reference genome (USMARCv1.0) using the STAR aligner, and quantified read counts for all genes annotated (USMARCv1.0) with HTSeq-count, version 0.5.3p9as previously reported^7^. Differential gene expression analysis was performed at GENEWIZ with the Bioconductor package DESeq.

### Polar metabolites LC/MS–MS

Polar metabolites were extracted from 10 mg flash-frozen tissue and 0.5 mL serum sample with 1 mL of ice-cold 80% (v/v) methanol and 0.6 mL acetonitrile and (200 µg of protein per sample) was analyzed using a 5500 QTRAP hybrid triple quadrupole mass spectrometer (AB/SCIEX) coupled to a Prominence UFLC HPLC system (Shimadzu) with SRM as in^7^. Peak areas from the total ion current for each metabolite SRM transition were integrated using MultiQuant v2.0 software (AB/SCIEX). LC/MS–MS was conducted for the individuals pig samples (eight pigs in eight independent runs). All analyses are conducted in agreement with the biosafety regulations at the Rhode Island Hospital, Providence, RI.

### Proteomic LC/MS–MS

100 mg of left ventricular myocardial tissue was lysed in lysis buffer (8 M urea, 1 mM sodium orthovanadate, 20 mM HEPES, 2.5 mM sodium pyrophosphate, 1 mM β-glycerophosphate, pH 8.0, 20 min, 4 °C). 200 µg of protein per sample was subjected for trypsin digestion. Peptide’s desalting was conducted on a C18 Sep-Pak cartridge Waters, MA), lyophilized, and dissolved in 0.1 M CH3COOH. LC/MS–MS was conducted on a fully automated proteomic technology platform that includes an Agilent 1200 Series Quaternary HPLC system (Agilent Technologies, Santa Clara, CA) connected to a Q Exactive Plus mass spectrometer (Thermo Fisher Scientific, Waltham, MA). (Agilent Technologies, CA) and Q Exactive Plus mass spectrometer (Thermo Fisher Scientific). The peptides were separated through a linear reversed-phase 90 min gradient from 0 to 40% buffer B (0.1 M acetic acid in acetonitrile) at a flow rate of 3 µL/min through a 3 µm 20 cm C18 column (OD/ID 360/75, Tip 8 µm, New objectives, Woburn, MA) for a total of 90 min run time. The electrospray voltage of 2.0 kV was applied in a split-flow configuration, and spectra were collected using a top-9 data-dependent method. Survey full-scan MS spectra (m/z 400–1800) were acquired at a resolution of 70,000 with an AGC target value of 3 × 106 ions or a maximum ion injection time of 200 ms. The peptide fragmentation was performed via higher-energy collision dissociation with the energy set at 28 normalized collision energy (NCE). The MS/MS spectra were acquired at a resolution of 17,500, with a targeted value of 2 × 104 ions or maximum integration time of 200 ms. The ion selection abundance threshold was set at 8.0 × 102 with charge state exclusion of unassigned and z = 1, or 6–8 ions and dynamic exclusion time of 30 s. Peptide spectrum matching of MS/MS spectra of each file was searched against the NCBI *Sus scrofa* database (TaxonID: 9823, downloaded on 11/21/2019) using the Sequest algorithm within Proteome Discoverer v 2.3 software (Thermo Fisher Scientific, San Jose, CA). The Sequest database search was performed with the following parameters: trypsin enzyme cleavage specificity, two possible missed cleavages, 10 ppm mass tolerance for precursor ions. Peptide assignments from the database search were filtered down to a 1% FDR. The relative label-free quantitative and comparative among the samples were performed using the Minora algorithm and the adjoining bioinformatics tools of the Proteome Discoverer 2.3 software. To select proteins that show a statistically significant change in abundance between two groups, a threshold of 1.5-fold change with p-value (0.05) were selected.

The comparative label-free relative quantitative proteomic analysis was performed for 6 samples (LD-3 pigs; MetS-3 pigs. A total of 89,349 peptide spectral match (PSM) was generated from 15,243 unique peptides corresponding to 2553 unique proteins identified by LC–MS/MS analysis. This unique protein set (2553) was further subjected for label free quantitative analysis: abundance at least 1.5-fold with significant p-value (≥ 0.05).

### Statistical analysis

Data analysis was performed using Microsoft Excel and Graphpad Prism7 software. As only 2 groups (LD and MetS) were compared, an unpaired, 2-tailed *Student t* test was applied to compare LD and MetS data sets. In all cases, p ≤ 0.05 was considered to be a statistically significant difference. Data are presented as means ± SD. Functional biological data analysis is conducted with the list of genes that are differentially expressed (p-values < 0.05) in response to diet using the g:Profiler approach^50^.

## Supplementary Information

Supplementary Information.

## Data Availability

The RNA-Seq data are available under GEO accession number PRJNA544355. The 51 targeted polar metabolites distribution in MetS and LD tissue and blood are shown in the supporting information (Table S1). The proteomics LC/MS–MS data are available upon request.
