# Author Correction: The cardiac molecular setting of metabolic syndrome in pigs reveals disease susceptibility and suggests mechanisms that exacerbate COVID-19 outcomes in patients

**DOI:** 10.1038/s41598-021-00995-z

**Published:** 2021-10-28

**Authors:** Olivia Ziegler, Nivedita Sriram, Vladimir Gelev, Denitsa Radeva, Kostadin Todorov, Jun Feng, Frank W. Sellke, Simon C. Robson, Makoto Hiromura, Boian S. Alexandrov, Anny Usheva

**Affiliations:** 1 Division of Cardiothoracic Surgery, Department of Surgery and, The Warren Alpert Medical School, Brown University, Providence, RI 02903 USA; 2 Beth Israel Deaconess Medical Center, Harvard Medical School, Boston, MA 02115 USA; 3 Department of Chemistry, Sofia University, Sofia, Bulgaria; 4 Medical University, Sofia, Bulgaria; 5 Daiichi University of Pharmacy, Fukuoka, 815-8511 Japan; 6 Los Alamos National Laboratory, Los Alamos, NM 87545 USA

Correction to: *Scientific Reports* 10.1038/s41598-021-99143-w, published online 05 October 2021

The original version of this Article contained an error in the spelling of the author Frank W. Sellke which was incorrectly given as Frank W. Selke.

The original Article has been corrected.

