# Supplementary material for: The cardiac molecular setting of metabolic syndrome in pigs reveals disease susceptibility and suggests mechanisms that exacerbate COVID-19 outcomes in patients
Source: Sci Rep. 2021 Oct 5;11:19752. doi: 10.1038/s41598-021-99143-w (PMC8492658; doi:10.1038/s41598-021-99143-w)
Supplement: Supplementary file 1 — Supplementary Information. [file 41598_2021_99143_MOESM1_ESM.pdf]

The cardiac molecular setting in Metabolic Syndrome highlights disease susceptibility and potential weakness in SARS-CoV-2 infection.

Authors: Olivia Ziegler [1], Nivedita Sriram[1], Vladimir Gelev[2,3], Denitsa Radeva[3], Kostadin Todorov[2,4], Jun Feng [1], Frank W. Selke[1], Simon C. Robson [2], Makoto Hiromura[5], Boian S. Alexandrov [6], Anny Usheva [1, #]

Institution and Affiliations: [1]Division of Cardiothoracic Surgery, Department of Surgery and, The Warren Alpert Medical School, Brown University, Providence, RI 02903, United States, [2]Beth Israel Deaconess Medical Center, Harvard Medical School, Boston MA 02115, United States, [3]Department of Chemistry, Sofia University, Sofia, Bulgaria, [4]Medical University, Sofia, Bulgaria, [5]Daiichi University of Pharmacy, Fukuoka, 815-8511, Japan, [6]Los Alamos National Laboratory, Los Alamos, NM 87545, United States.

Figure S1. g:GOSt multiquery diagram of pathways with differential response to diet. g:GOSt multiquery diagram summarizes results from RNA-seq. The data sources that are used to identify pathways in the myocardial response to diet are shown at left: BP-gene ontology; biological pathway: KEGG, REAC – Reactome, WP- WikiPathways, HP-Human phenotype ontology. The term name column shows the individual pathways; Term ID-the individual pathways ID; p_{adj} (<0.05) –adjusted p-value for the association of the pathways with the differential response to diet. Selected pathways in the diagram are with highest significance ($p_{adj} < 1e^{-4}$) in the corresponding data source; the bar diagrams on right show the negative logarithm of the p_{adj} value. The $-\log_{10(padj)}$ axis is shown at the top of the bar diagrams and signifies the pathway -the strongest associations have smallest p_{adj} values and their negative logs will be the greatest-bar color from dark green to yellow (0 – 16 $-\log_{10(adj)}$).

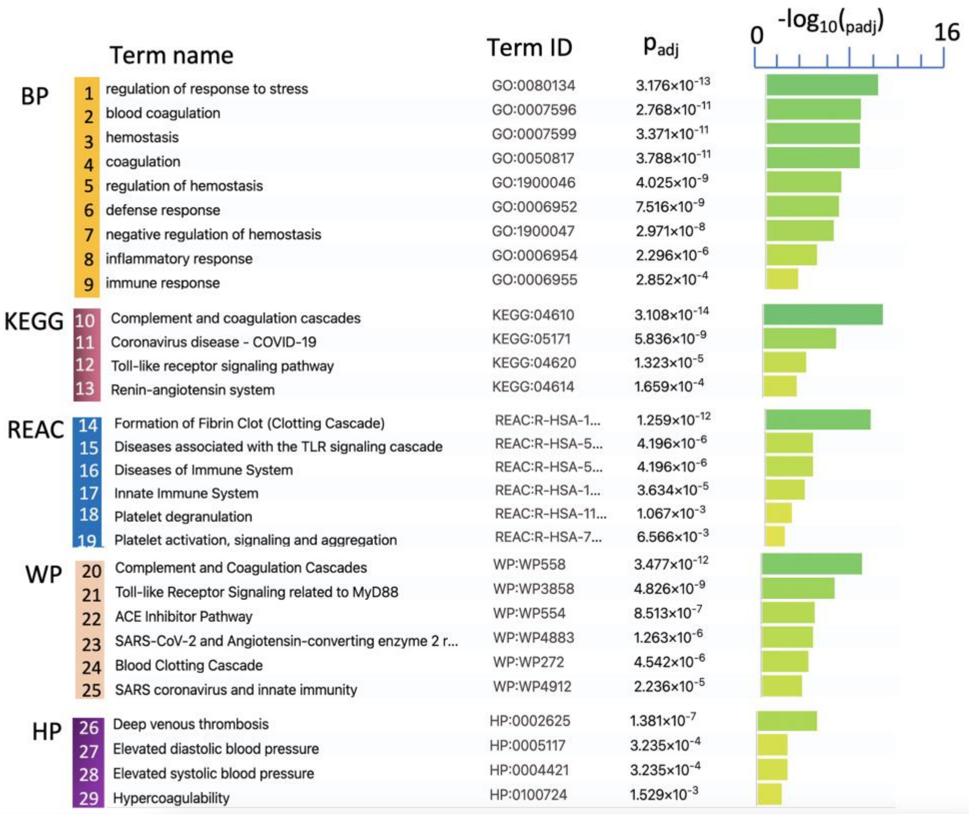

Figure S2 (related to Fig. 2a,b, Fig.4a, Fig.5a) Control genes and metabolites that do not have a significant change in LD and MetS. RNA-seq, proteomics and targeted polar metabolites LC/MS-MS were applied to identify and compare contents of gene products and metabolites. Myocardial mRNA, proteomic, and targeted polar metabolites libraries from LD (n=4) pigs and MetS (n=4) pigs are compared for relative content of genes and metabolites. Parallel blood samples LD (n=4) and MetS (n=4) are used to perform metabolomic analyses. (**a**) Genes that do not change significantly in response to diet at mRNA level. The abundantly expressed genes are compared at both protein and mRNA levels : desmin, ACTA1, ACTC2, VWF, METTL1, P2RY6, P2RY12, SERPING1, C1QBP, C1QA. Values are in relative units (R.U.); as shown on the verticals; means +/-SD, P<0.05. (**b**) the polar metabolites hydroxyphenylpyruvate, N-Acetyl-L-alanine do not respond to diet in tissue. **(c)** Metabolites that are highly abundant in the blood vs tissue in both LD and MetS: D-glyceraldehdye-3-phosphate, ascorbic acid. **(d)** The D-sedoheptulose-1-7-phosphate is nearly identical in tissue and blood, with no reaction to diet. **(e)** Western blot reaction with antibody to CD36 and total tissue lysates (50 ug protein) from 3 LD pigs lines 1, 2, 3 and 3 MetS pigs lines 4, 5, 6. The genes, proteins, and metabolites are shown at the top of the diagrams. The color identity of the bars is shown at the bottom . Values are in relative units; all bar diagrams are mean +/-SD, P<0.05. 200 µg of protein per tissue and blood sample was subjected to proteomic and metabolomic LC/MS-MS. RNA-seq is performed with 50 mg total RNA per tissue sample.

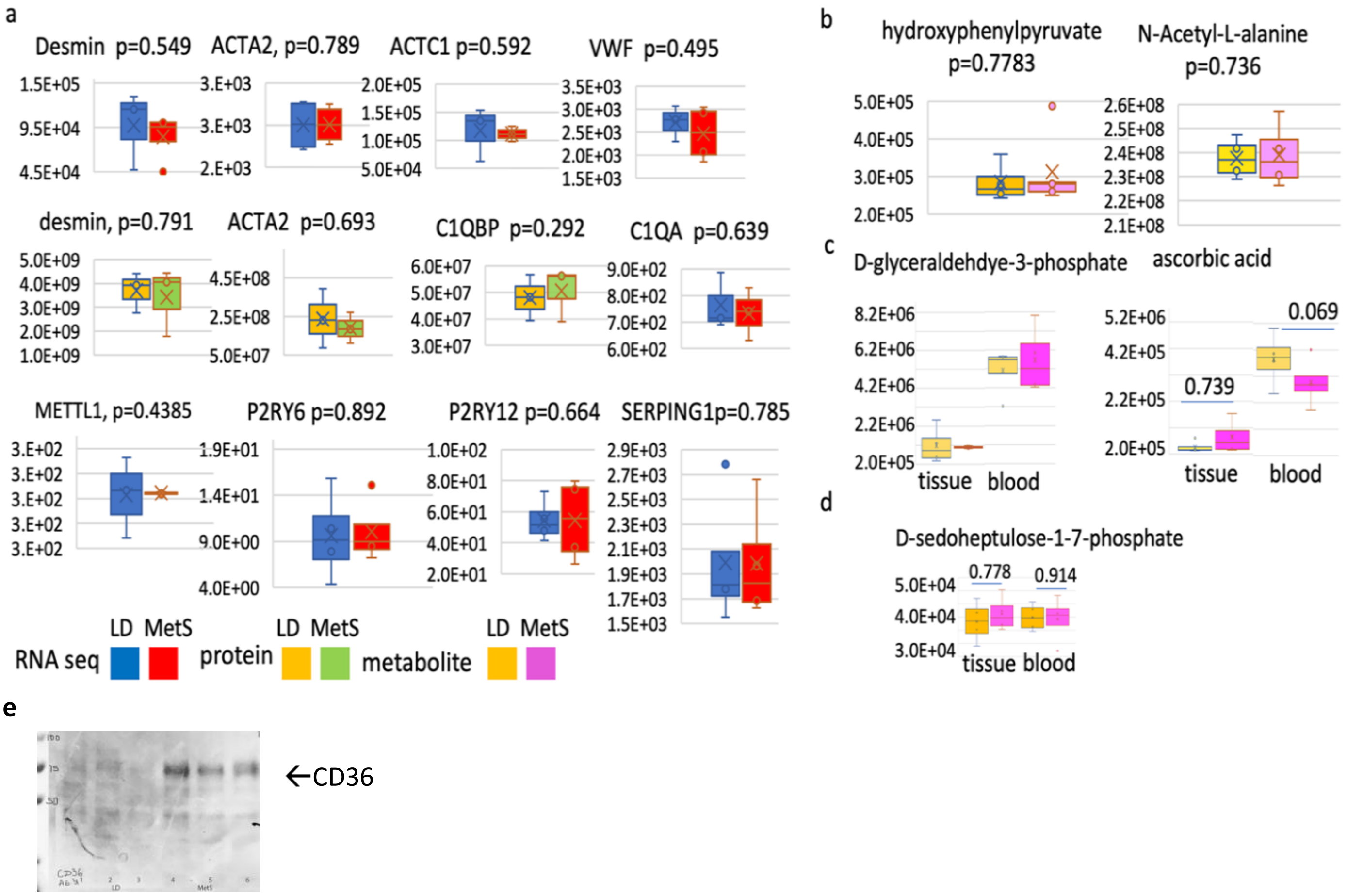

Table S1 (related to fig. 2b, 4b, 6a) Simultaneous quantification of polar metabolites in myocardial tissue and blood and in response to diet. The name of the metabolites is in the right column; metabolites weigh (relative units) in LD tissue is gated in blue; gray, MetS tissue; yellow, LD blood; orange, MetS blood as shown at the top

of the columns. LC/MS-MS was run for the individual pig samples (16 independent runs). MultiQuant v2.0 software (AB/SCIEX) was used for peak areas integration [1]. LC/MS-MS was conducted as previously reported[9]. 200 µg of protein per tissue and blood sample was subjected to LC/MS-MS.

ple ID	iac tissue LD				iac tissue MetS				d LD				d MetS			
	1	2	3	4	5	6	7	8	1	2	3	4	5	6	7	8
droxy-acetone-phosphate	1719305.71	2978269.29	3645483.83	2026629.69	3380020.84	3387982.76	2287900.08	2752506.56	7355110.15	6813660.58	4934227.18	5439965.83	10250920.5	5391965.22	5803703.85	269.51
yceraldehdye-3-phosphate	571197.135	2537422.17	1258021.02	386440.694	1057229.9	1183270.91	1138557.78	1025867.09	5807406.84	5856482.53	3227129.71	5545725.27	8017351.23	4230109.92	6055324.64	475.75
ycerol-3-phosphate	98534459.9	237097117	233718287	159955224	194882877	234636814	149742754	237767661	1688987	587877.109	1570911.66	34379.3274	3004255.25	3229440.24	859767.897	453.16
rbic acid	366926.518	326558.879	803804.294	354477.586	354940.985	388940.565	878564.346	1740827.85	5008844.65	4053236.54	3764909.64	2541552.41	2890646	4206572.6	1923875.71	323.09
propylmalic acid	986277.823	6442331.17	561673.667	681075.726	589058.341	856086.981	612425.214	663104.056	509236.46	595971.165	236188.856	140472.302	136949.886	192463.322	142667.921	01.668
rbamoyl-L-aspartate-nega	1112619.12	312091.067	121706.654	469169.355	398744.717	368422.404	776008.516	210970.304	1117295.64	439275.636	561806.286	410289.088	275472.121	485107.614	179475.752	38.712
phosphate	4133325.63	10796062.3	10307168.2	4494394.78	7476039.35	20316136.8	15085851	14131518.9	5009844.9	1643178.34	11335661.8	9795708.24	13278048.9	16181160.4	1180159.86	677.27
xylate	1133801.8	955229.682	703144.162	1104793.95	110999.096	81376.5415	77984.1886	135151.313	315016.278	224131.708	349401.751	176776.314	326361.928	420819.063	133393.572	06.534
olate	58742.476	102678.757	69201.0935	149066.315	227029.864	225333.763	280785.808	202023.931	13802.4142	136047.561	264967.079	300835.962	268988.985	254020.995	55862.5039	5.7876
vate	390155.5	337051.172	520714.872	496241.725	219818.734	185347.777	167793.735	139378.242	100296.943	25680.3061	118348.779	121193.856	122838.535	125680.267	54294.3701	3.4866
te	548803754	336365977	293636852	219459682	159204076	86140145.4	138603208	59692836.8	106361476	81552525.8	104868781	108597507	108813176	106342968	68009160.4	7335.5
obutanoate	564846.35	1445412.09	45621.483	67073.1096	508316.569	1052125.49	367637.063	1093055.34	112102.187	51002.998	561278.272	267049.37	124754.549	443383.131	41401.3523	.8.3842
pacetate	415775.373	170891.241	308124.362	80212.5018	518509.55	1091214.01	393560.847	847898.438	470970.81	453256.536	368341.562	245078.303	251516.861	337185.246	266023.431	52.762
erate	319239.481	220321.062	298994.384	335400.067	478625.176	761471.485	729141.284	477841.995	272455.657	135271.726	956431.365	529255.659	799090.257	822895.697	81144.2392	47.499
il	4278406.48	56090491.2	54305646.4	12973143.9	52116979.7	36184316.8	49286635.7	48496968.4	2546505.22	1511279.62	11808488.2	11073816.8	5450277.12	5802282.5	1951846.37	604.32
arate	42768884.2	46178803.4	43908413.7	81464110.6	28295365.7	15060452.6	21643890.8	36294487.9	865061.613	704082.639	1075072.69	901275.509	927952.551	1324264.47	949022.256	087.42
eic acid	12421038.2	45008491.5	42062614.9	19910806.2	28708189.7	43602211.6	20499883.1	34499697.3	890356.927	758589.633	1095372.04	692587.128	1072266.91	1281607.72	872526.337	661.62
oxyphenylpyruvate	359388.396	317449.061	279556.28	242793.295	254175.035	250624.218	259243.34	486405.624	289243.224	218825.548	178626.112	342178.67	235067.852	215945.272	426064.543	288120.028
ose-1-phosphate	1553970.8	3879582.23	4887181.94	1345365.07	1299219.94	5929832.56	945784.356	1619102.26	526318.414	770470.088	318654.888	243538.203	289572.049	319626.053	61939.0317	168340.936
ose-6-phosphate	8496756.94	8979202.64	6030391.02	6209986.79	1364920.61	2008756.83	1157920.87	2137014.71	243113.111	54226.7305	373067.457	303738.809	383568.402	221222.924	43472.9926	107264.097
tose-6-phosphate	5299433.67	7810357.58	5009180.17	7529100.39	2478004.44	3914304.15	767797.123	1536457.49	361695.537	167877.803	472970.703	505026.512	480311.208	298378.157	54618.5208	144397.717
diphopshateglycerate	355634.494	378797.871	282662.847	765343.261	214265.908	268960.666	210531.822	225683.382	310303011	173809252	338748742	329458830	322762970	372991809	90316487.4	296821539
Diphosphoglyceric acid	47700.9761	30917.5038	91884.5667	759525.718	66824.4528	83311.6355	72422.6783	74496.6311	317189938	171755642	323510111	334521098	336757731	391786988	96142421.7	305501635
osyl-L-homocysteine-nega	125146.196	194222.992	29168.5484	39160.5523	165327.752	137654.061	466331.371	236969.764	30087.744		59734.2909	38805.9524	36446.5089	38718.1049	8197.81453	11370.9355
ne	133438803	212640404	214011432	203754961	207561441	210766232	211505970	207653962	21473804.3	4043237.4	8870807.68	7948438.35	6190738.14	21306293.9	1232846.79	3063828.69
ospho-D-gluconate	743404.318	3899503.15	2565048.28	256928.091	604716.682	929931.579	1063903.86	789206.34	417238.804	226256.169	1122513.36	1210461.59	1261596.57	1297913.65	91864.7306	475264.331
hosine	317698.807	1481691.82	1532826.4	856671.796	723710.342	1458655.5	468303.571	1222466.7	307352.19	184998.976	242394.348	155253.125	189779.503	253536.398	254169.239	562770.865
doheptulose-1-7-phosphate	52129.0327	33981.5567	40238.1568	46759.4521	40297.3912	47376.52	55350.1375	42147.506	39563.8837	50667.2208	41359.1423	47952.1252	45021.1611	32204.1446	53268.8274	46494.162
etyl-glucosamine-1-phosphate	981225.351	2140888.63	1778076	1184563.17	277392.055	673420.804	1258904.74	980495.637	352357.247	162644.214	428512.362	12761.8318	460278.764	554950.87	197029.232	387014.027
pacetate	936666.263	956892.85	1071482.66	1091773.21	694256.319	528465.969	377080.262	492642.087	926036.58	1225468.28	1061749.95	1072876.25	951620.137	892952.795	1089182.11	1010817.83
pacetate	936666.263	956892.85	1071482.66	1091773.21	694256.319	528465.969	377080.262	492642.087	926036.58	1225468.28	1061749.95	1072876.25	951620.137	892952.795	1089182.11	1010817.83
te	143303539	159446669	149286887	82314138.5	59859604.4	38380466.5	49403161.2	69774536.9	5074504.32	4229125.09	7130833.47	5586474.26	6301239.45	8061748.35	6273857.02	7958717.32
xanthine	90604179.3	147860839	71258143.1	102862688	26846889.8	59125534.6	61404179.3	42097734.5	9324081.96	8364169.82	8987524.98	9402783.48	10161873.3	9695925.77	7616905.91	9616707.37
toglutarate	23679559.8	29181258.8	32747898.5	32776832.5	21470236.7	22110861.6	22019401.7	21059268.5	2226531.18	937469.651	5835443.37	2267497.47	5717445.21	6008737.11	1052853.56	2781387.75
olinate	305214.499	79277.1696	70401.2233	137027.613	218659.942	143603.35	249842.27	119204.44	754036.704	463172.164	551333.765	442053.009	649941.031	798703.735	563693.523	826289.322

acid	99582.5069	112036.439	95545.0191	72167.5141	199534.223	169256.237	245771.831	239674.156	53455.8185	23284.4158	63894.5076	135097.723	43248.8494	69623.9471	12489.4078	34221.1936
ono-?-lactone	25467.9456	15507.1777	34893.2167	29746.203	11811.801	27806.2286	40671.5238	36063.5147	34409.6203	11043.6069	30557.3184	22166.2537	70725.7188	102988.567	49371.3269	105587.097
-inositol	245206557	293085957	231437429	259434900	265875033	317036474	308574139	279369519	599978.326	375048.262	22629246.6	23466201.6	6754395.72	4755280.98	1443042.03	6660995.54
ocysteic acid	612505.445	920836.752	1601723.72	223394.519	3338226.31	1313845.33	2515189.62	2074130.26	241665.081	114851.969	493544.387	483258.267	4879.72256	499195.097	89986.5439	329359.563
c-AMP	18307634.2	11902620.7	7530565.82	22517128.3	3258370.66	1883130.57	2553531.62	1408896.64	108774.732	30816.7646	120663.414	180127.021	116083.319	144353.595	74960.7933	95057.1736
enosyl-L-methionine	6009297.41	8955664.6	9937043.49	10513852.4	17293345	32096836.3	28768837.7	16338687.3	469358.63	873283.29	637622.204	222480.146	293631.996	626848.851	406532.265	412148.287
nine	44410255.4	48747222.9	74504319.4	36361481.6	86255881.6	150395462	120896535	112879097	24086978.2	30196261.5	67134676.4	45335564	61334301.2	53689618	2389597.62	44643151.3
lline	33427850.4	50985023.1	80020639.8	54689546.6	8472242.2	8084365.41	7707564.42	1660191.44	18943853.1	8087232.33	12152956.8	6090968.39	66065868.1	67127998.9	11103277	30953507
IG-dimethyl-L-arginine	19553692.6	17099824.1	32605035	10113569	45054968.5	76677941.3	113119201	80837515.8	8603223.91	4564767.72	35147520.6	27657647.1	63756197.4	43667821.8	3924277.46	12409309.6
thine	3339637.52	3015992.52	6955082.45	5859766.18	20476487.3	19718612.4	13875733.3	28614108.2	9649579.94	4915698.8	49982676.3	49993456.4	55127118.5	52376607	2221884.31	14918323.9
	35499025.3	27416849	31798706.9	16451068	48170303.6	61288702.9	83380027.8	37698102.4	26789667.3	14795250.5	24725624.9	20209074.6	31104900.1	23958986.2	3624687.23	22052757
	99082445.8	72514511.2	41729241.7	196483360	4086276.8	37181364.1	12632048.7	8980971.58	85685324.3	23741575.2	72423821.9	101539996	11532547	10350748.5	10467365.1	14900596.9
c-AMP	18307634.2	11902620.7	7530565.82	22517128.3	3258370.66	1883130.57	2553531.62	1408896.64	108774.732	30816.7646	120663.414	180127.021	116083.319	144353.595	74960.7933	95057.1736
enosyl-L-homocysteine	54133.4879	57371.6043	42430.276	37817.9947	82047.6363	101595.736	111476.191	107368.991	23010.2348	32006.7355	37238.5578	30600.1576	20176.5794	27699.3157	41093.1314	28313.5691
ocystein	21133.0112	35371.37	29430.276	31137.88	81811.23	72595.32	72562.1132	101416.54	9010.1109	6813.5621	8391.63009	11083.61	21003.83	25687.4481	49178.1413	29112.9521
etyl-L-alanine	227830441	241665268	229041981	247382817	232346336	230714412	257085922	241443622	29251115.6	14287981.7	85741653	38532534.9	70733534.2	73318938.4	9528068.85	23345905.3

1. Yuan M, Breitkopf SB, Yang X, Asara JM. A positive/negative ion-switching, targeted mass spectrometry-based metabolomics platform for bodily fluids, cells, and fresh and fixed tissue. *Nat. Protoc.* **7**:872–881. doi: 10.1038/nprot.2012.024 (2012).
